# Genomic mating in outbred species: predicting cross usefulness with additive and total genetic covariance matrices

**DOI:** 10.1093/genetics/iyab122

**Published:** 2021-09-03

**Authors:** Marnin D Wolfe, Ariel W Chan, Peter Kulakow, Ismail Rabbi, Jean-Luc Jannink

**Affiliations:** 1 Section on Plant Breeding and Genetics, School of Integrative Plant Sciences, Cornell University, Ithaca, NY 14850, USA; 2 International Institute of Tropical Agriculture (IITA), Ibadan, Nigeria; 3 USDA-ARS, Ithaca, NY 14850, USA

**Keywords:** genomic mate selection, variance prediction, directional dominance, nonadditive effects, cassava, Genomic Prediction, GenPred, Shared Data Resources

## Abstract

Diverse crops are both outbred and clonally propagated. Breeders typically use truncation selection of parents and invest significant time, land, and money evaluating the progeny of crosses to find exceptional genotypes. We developed and tested genomic *mate* selection criteria suitable for organisms of arbitrary homozygosity level where the full-sibling progeny are of direct interest as future parents and/or cultivars. We extended cross variance and covariance variance prediction to include dominance effects and predicted the multivariate selection index genetic variance of crosses based on haplotypes of proposed parents, marker effects, and recombination frequencies. We combined the predicted mean and variance into usefulness criteria for parent and variety development. We present an empirical study of cassava (*Manihot esculenta*), a staple tropical root crop. We assessed the potential to predict the multivariate genetic distribution (means, variances, and trait covariances) of 462 cassava families in terms of additive and total value using cross-validation. Most variance (89%) and covariance (70%) prediction accuracy estimates were greater than zero. The usefulness of crosses was accurately predicted with good correspondence between the predicted and the actual mean performance of family members breeders selected for advancement as new parents and candidate varieties. We also used a directional dominance model to quantify significant inbreeding depression for most traits. We predicted 47,083 possible crosses of 306 parents and contrasted them to those previously tested to show how mate selection can reveal the new potential within the germplasm. We enable breeders to consider the potential of crosses to produce future parents (progeny with top breeding values) and varieties (progeny with top own performance).

## Introduction

Diverse crops ranging from staples (*e.g.*, cassava and potato) to cash crops (*e.g.*, cacao) to forestry products (*e.g.*, eucalyptus) are both outbred and clonally propagated (Gemenet and Khan 2017). In these crops, exceptional genotypes can be immortalized and commercialized as clonal varieties. Few clonal crops are also inbred thus, like livestock, each cross segregates phenotypically to different degrees. Unlike seed crops (*e.g.*, maize, wheat), inbreeding is unnecessary for product development. Consider a breeding program implementing some form of genomic selection (GS) (Heffner *et al.* 2009; Jannink *et al.* 2010) on a population. All extant members and future progeny are or will be genotyped using genome-wide markers. Field evaluations are conducted at species- and trait-appropriate stages for one or more traits, on at least a subset of the genotypes. Genomic prediction is used to increase selection intensity and decrease cycle times by providing selection criteria for more genotypes, faster than would have otherwise been possible (Hickey *et al.* 2017). The breeding scheme can be further divided into two parts (Gaynor *et al.* 2017; Santantonio and Robbins, 2020; Werner *et al.* 2020) consisting of (1) population improvement by recurrent selection (RS) and (2) a variety development pipeline (VDP). RS is done in order to manage and improve the frequency of beneficial alleles in the population over time. The VDP consists of a series of field trials in which candidates’ performance is evaluated. For clonal crops, germplasm is advanced from one VDP stage to the next by vegetative propagation.

### The importance of matings and the need for mate selection criteria

Every cross is important. Crosses imply an opportunity and a risk. New matings generate genetic variation, the substrate on which selection can operate. However, for a breeder, new crosses require investment of time, land, and money, especially considering the added costs of genotyping. Moreover, crosses may exhibit inbreeding depression or heterosis. Thus, matings serve the multiple purposes of producing new candidate breeding parents for RS and/or cultivars for VDP, potentially evaluated for multiple product profiles characterized by unique selection indices (SI). Selection to drive improvement in the population’s mean over time to meet the objective of RS centers on allele-substitution effects and the breeding value (BV). For the VDP, selecting clones to advance for testing should be based on the total genetic value (TGV) of an individual which includes nonadditive genetic effects such as dominance.

### Genomic predictions incorporating nonadditive effects

Nonadditive effects can be included in genomic predictions in a number of ways (Vitezica *et al.* 2013; Varona *et al.* 2018). Most of the literature so far has dealt with including nonadditive effects in the prediction of the genetic values of an existing pool of selection candidates (Varona *et al.* 2018). Nonadditive predictions have often been shown to increase prediction accuracy (Heslot *et al.* 2012; Wolfe *et al.* 2016a; Werner *et al.* 2020). In addition, the mean performance (mean TGV) of the progeny can deviate from the prediction based on the mean BV of the parents in the presence of nonadditive effects. Genomic predictions of cross mean TGV have been applied to hybrid performance (Alves *et al.* 2019) and mate allocation (Toro and Varona 2010). Predictions can also include genome-wide inbreeding/overdominance effects, also referred to as directional dominance; Xiang *et al.* (2016) and this has recently been shown to be advantageous in a simulated two-part clonal crop breeding scheme (Werner *et al.* 2020).

### Genomic mate selection for outbred, clonal crops

When one or both parents are heterozygous, offspring are expected to segregate for their BV and TGVs. The relative advantage of possible pairwise matings can best be distinguished when predictions of both the genetic mean *and* variance are available. The usefulness criterion (UC) or simply “usefulness” of a cross is a prediction of the mean performance of the selected superior fraction of the progeny: UC=μ+i×σ, where *σ* is the predicted genetic standard deviation of the progeny and *i* is the standardized selection intensity (Zhong and Jannink 2007; Segelke *et al.* 2014; Lehermeier *et al.* 2017b). The additive genetic variance of an infinite pool of progeny from a cross can be predicted deterministically using the combination of genome-wide marker effects, a genetic map, and phased parental haplotypes (Lehermeier *et al.* 2017b). This approach has almost exclusively been applied to the prediction of additive genetic variance and covariance (Neyhart *et al.* 2019). Bonk *et al.* (2016) showed that dominance in addition to additive within-family variances can be deterministically predicted in outbred species based on gametic variances of putative parents (Bijma *et al.* 2020). Most other applications are predictions of the variance of inbred lines derived from inbred founders (Zhong and Jannink 2007; Lehermeier *et al.* 2017b; Allier *et al.* 2019b; Neyhart *et al.* 2019; Neyhart and Smith 2019).

### Criteria and methods developed in this study

In this study, we extend the deterministic prediction of progeny variances in several ways to maximize the utility and practicality of implementing genomic mate selection. First, we show how to include dominance in the prediction of cross genetic variance and we do so for founders of arbitrary inbreeding level. Next, we distinguish two types of cross usefulness: usefulness for RS (*i.e.*, the predicted mean BV of offspring selected as parents; *UC_parent_*) and usefulness for variety development (*i.e.*, the predicted mean TGV of clones advanced as varieties in the VDP; *UC_variety_*). Finally, since matings are usually chosenbased on multiple traits, we extend the prediction to cross variance on SI. We show that to predict index variance, we must predict the full matrix of trait genetic variances and covariances (Bonk *et al.* 2016; Allier *et al.* 2019b; Neyhart *et al.* 2019). We implement the core functions for multi-trait prediction of outbred cross variances including additive and dominance effects in an R package predCrossVar.

### Empirical study of cassava

We present an empirical study of the accuracy for predicting additive and nonadditive genomic mate selection criteria. We set up a cross-validation scheme that measures the accuracy of predicting means, variances and usefulnesses of previously untested crosses using data from a real cassava (*Manihot esculenta*) breeding program. Cassava is one of the most important tropical staple foods, especially in Africa (http://faostat.fao.org). Among outbred, clonal crops, GS is relatively mature in cassava breeding (de Oliveira *et al.* 2012; Ly *et al.* 2013; Wolfe *et al.* 2016a,b, 2017; Elias *et al.* 2018; Yonis *et al.* 2020; Okeke *et al.* 2017; Ozimati *et al.* 2018) because of the Next Generation Cassava Breeding Project (http://www.nextgencassava.org, est. 2012), and the species can serve as a model for many others. We leverage a validated GS pedigree with genome-wide phased haplotypes and a genetic map (Chan *et al.* 2019). We used a directional dominance model (Xiang *et al.* 2016) to make first-time estimates of genome-wide inbreeding (homozygosity) effects in cassava. We report our empirical study in a fully reproducible and documented framework (https://wolfemd.github.io/PredictOutbredCrossVar/).

## Methods

### Formulation of genomic predictions and selection criteria

Below, we describe predictions that are applicable as selection criteria, first for genomic truncation selection *G_TS_*, followed by extensions that enable mate selection *G_MS_*. Throughout, we distinguish selection criteria based on their suitability for evaluating the potential of individuals (for *G_TS_*) or crosses (for *G_MS_*) for RS *vs* VDP.

### *G_TS_*: Selecting genotypes with predictions about generation *t*

Genomic recurrent TS (*G_TS_*) evaluates existing individuals, either for their potential as parents (without regards to specific mates) and/or their potential as clonal cultivars. Under a nonepistatic model, the TGVs of individuals in the current population (time t) can be partitioned into a BV (*g_BV_*) and a dominance deviation *g_DD_*.

$$g_{\mathrm{TGV}}=g_{BV}+g_{DD}$$

Consider a diploid population with *n* individuals genotyped at *p* biallelic genomic loci.

y=Xβ+Zα+Wd+ϵ

In this linear model, the n×1 vector of phenotypic observations, ***y*** is modeled according to a combination of genetic and nongenetic effects. Fixed experimental design-related effects estimates are given by β and its corresponding incidence matrix ***X***($\left[n\times N_{\mathrm{fixed}}\right]$) where *N*_fixed_ is the number of fixed factors. The elements of the [n×p] matrices ***Z*** and ***W*** contain column-centered marker genotypes:

$$\begin{array}{c} \begin{array}{c} z_{ij}=\{\begin{array}{cc} 2-2p_{j} & A_{1}A_{1}\\ 1-2p_{j} & A_{1}A_{2}\\ 0-2p_{j} & A_{2}A_{2} \end{array} \end{array}\\ \begin{array}{c} w_{ij}=\{\begin{array}{cc} -2q_{j}^{2} & A_{1}A_{1}\\ 2p_{j}q_{j} & A_{1}A_{2}\\ -2p_{j}^{2} & A_{2}A_{2} \end{array} \end{array} \end{array}$$

Here, *p_j_ and q_j_* are the population allele frequencies, as opposed to the within-parent allele frequencies, which are referred to later on. This encoding of genotypes results in marker effects (α and ***d***) that correspond to allele substitution and dominance deviation effects (Vitezica *et al.* 2013). The marker effects can then be used to predict genomic estimated TGVs (**GETGV**, ${\hat{g}}_{\mathrm{TGV}}$) as the sum of the genomic estimated BV (**GEBV**, ${\hat{\text{g}}}_{BV}=\text{Z}\hat{\alpha }$) and a corresponding dominance deviation (**GEDD**, ${\hat{g}}_{DD}=\text{W}\hat{\text{d}}$). The GEBV predicts the mean offspring of a clone mated at random and as such is suitable for truncation RS of parents. The GETGV predicts the performance of each clone, rather than any property of its offspring and is useful for selection for variety advancement.

### *G_MS_*: Selecting crosses with predictions about generation *t + 1*

GEBV and GETGV enable us to do truncation selection. In order to implement mate selection, criteria that distinguish crosses are needed. Progeny of crosses may segregate for both their breeding and TGVs. Crosses may thus differ in their likelihood of producing progeny that are superior varieties (high $g_{\mathrm{TGV},t+1}$) and/or parents (high $g_{BV,t+1}$). We focus here on distinguishing the best crosses on the basis of both their predicted genetic means *and* variances.

### Predicted cross means

The family mean, *μ_BV_* can be predicted as the mean of parental BVs.

$$\mu_{BV}=\frac{\mathrm{GEB}V_{P1}+\mathrm{GEB}V_{P2}}{2}$$

Dominance deviation can be included in order to predict the mean TGV, *μ_TGV_* according to Equation 14.6 (Falconer and Mackay 1996; Toro and Varona 2010; Varona *et al.* 2018; Werner *et al.* 2020).

$$\mu_{\mathrm{TGV}}=\sum_{k=1}^{p}a_{k}(p_{ik}-q_{ik}-y_{k})+d_{k}[2p_{ik}q_{ik}+y_{k}(p_{ik}-q_{ik})]$$

Here, *p_ik_ and q_ik_* are the allele frequencies of the counted (alternative) and the noncounted (reference-genome) allele, respectively, for one of the two parents (indexed by *i*). The difference in frequency between the parent one (indexed by *i*) and the parent two (indexed by *j*) is,$y_{k}=p_{ik}-p_{jk}$ and the summation is over the *p* markers indexed by *k*. Note that *a_k_* is the average effect and *not* the allele substitution effect, *α* estimated by the additive-dominance parameterization presented above. As a result, predicting *μ_TGV_* with the formula above may not be appropriate. We adopt a suitable additive-dominance partition, described below, in our primary analyses.

### Predicted cross variances

The within-cross additive genetic variance can be predicted deterministically, relying on the formula for the genetic variance under linkage disequilibrium using Equation 5.16a (Lynch and Walsh 1998; Lehermeier *et al.* 2017b). Below, we use Equation 5.16b (Lynch and Walsh 1998) to predict dominance variance deterministically in an infinite population of diploid full-siblings (Bonk *et al.* 2016).

$${\hat{\sigma }}_{BV}^{2}=\alpha^{T}D\alpha$$

 ${\hat{\sigma }}_{DD}^{2}=d^{T}D^{2}d$, where $D^{2}=D⊙D,\;⊙$ indicating element-wise (Hadamard) multiplication of ***D***, having the effect of squaring all elements.

$${\hat{\sigma }}_{\mathrm{TGV}}^{2}={\hat{\sigma }}_{BV}^{2}+{\hat{\sigma }}_{DD}^{2}$$

The *p *×* p* variance-covariance matrix, ***D***, is the expected linkage disequilibrium among full-siblings by considering the expected pairwise recombination frequency and each parent’s haplotype phase.

$$\begin{array}{c} D_{P_{1}}^{\mathrm{gametes}}=(1-2c)⊙D_{P_{1}}^{\mathrm{haplos}}\\ D_{P_{2}}^{\mathrm{gametes}}=(1-2c)⊙D_{P_{2}}^{\mathrm{haplos}}\\ D_{P_{1}\times P_{2}}^{\mathrm{genotypes}}=D_{P_{1}}^{\mathrm{gametes}}+D_{P_{2}}^{\mathrm{gametes}} \end{array}$$

 $D_{P_{1}}^{\mathrm{haplos}}$ and $D_{P_{2}}^{\mathrm{haplos}}$ are simply the *p *×* p* covariance matrices associated with each parent’s respective 2×p haplotype matrix ($H_{P_{1}orP_{2}}$), where elements are 1 if the counted allele is present, 0 otherwise. We computed $D^{\mathrm{haplos}}=\frac{1}{2}H^{T}H-pp^{T}$, where ***p*** is a vector of within-individual, per-SNP allele frequencies (Alachiotis *et al.* 2016).

The *p *×* p* pairwise recombination frequencies matrix is **c** and can be derived from a genetic map. $D_{P_{1}}^{\mathrm{gametes}}$ and $D_{P_{2}}^{\mathrm{gametes}}$ are the covariance matrices for each parents pool of possible gametes, whose covariances sum to give the expected covariances genotypes in the cross, $D_{P_{1}\times P_{2}}^{\mathrm{genotypes}}$. The genetic variances ${\hat{\sigma }}_{BV}^{2}$ and ${\hat{\sigma }}_{DD}^{2}$ are thus predicted as above by using $D=D_{P_{1}\times P_{2}}^{\mathrm{genotypes}}$.

### Usefulness criteria (UC)—mean of superior family members

Given that predictions of genetic means and variances for a cross are available, they can be combined into a single cross selection criterion. We focus here on the UC, which predicts the mean (BV) of the superior progeny from a cross, *i.e.*, the mean *after* selection (Schnell and Utz 1975; Zhong and Jannink 2007; Lehermeier *et al.* 2017b). We note that predictions of cross means and variances may be used in other ways (Bijma *et al.* 2020), but focus on UC. The UC=μ+i×σ, where *μ* is the predicted mean of the cross, *i* is the standardized within-family selection intensity and *σ* is the predicted cross standard deviation.

In the context of the two-part breeding scheme for GS in clonal crops, crosses may be useful for producing both new parents and new varieties. We, therefore, define *e* therefore distinguish two UCs: $UC_{\mathrm{parent}}$and $UC_{\mathrm{variety}}$ (Table 1). Notice that in addition to separate predictions of mean and variance for $UC_{\mathrm{parent}}$  *vs* $UC_{\mathrm{variety}}$, two-part GS implies that the within-family intensity of selection for RS does not necessarily equal that of the VDP (Santantonio and Robbins 2020).

**Table 1:** Criteria for evaluating crosses

Parameter	Breeding values	Total genetic values
Mean	*μ_BV_*	*μ_TGV_*
Variance	$\sigma_{BV}^{2}$	$\sigma_{\mathrm{TGV}}^{2}=\sigma_{BV}^{2}+\sigma_{DD}^{2}$
Usefulness	$UC_{\mathrm{parent}}=\mu_{BV}+(i_{RS}\times \sigma_{BV})$	$UC_{\mathrm{variety}}=\mu_{\mathrm{TGV}}+(i_{\mathrm{VDP}}\times \sigma_{\mathrm{TGV}})$

### Extension to multi-trait selection indices

Parent selection is often done based on a multi-character selection index (SI). Crosses can be considered for their potential to produce progeny with good merit on one or more SI by first predicting the variances and covariances for each trait on the SI (Bonk *et al.* 2016; Allier *et al.* 2019b; Neyhart *et al.* 2019). We can therefore predict the mean and variance of a cross on the SI as follows:

1. Predict (co)variances for all traits on SI. Consider an index with two traits, *T*1 and *T*2.
  $$\sigma_{T1}^{2}=\alpha_{T1}^{T}D\alpha_{T1}$$
   
  $$\sigma_{T2}^{2}=\alpha_{T2}^{T}D\alpha_{T2}$$
   
  $$\sigma_{T1,T2}=\alpha_{T1}^{T}D\alpha_{T2}$$

Apply to dominance by substituting α with ***d*** and squaring elements of ***D***.

1. Compute the predicted mean and variance on the SI.
  $$\begin{array}{c} \mu_{SI}=w^{T}{\hat{g}}_{BV}\\ \sigma_{SI}^{2}=w^{T}\mathrm{Gw} \end{array}$$

The *n *×* T* matrix ${\hat{g}}_{BV}$ contains the GEBV for each trait and the T×1 vector ***w*** are the index weights. The *T *×* T* matrix ***G*** is the additive (or total) genetic variance-covariance matrix for traits on the index.

$$G=\left[\begin{array}{ccc} \sigma_{\mathrm{Trai}t_{1},\mathrm{Trai}t_{1}}^{2} & \ldots & \sigma_{\mathrm{Trai}t_{1},\mathrm{Trai}t_{T}}\\ \vdots & \ddots & \vdots \\ \sigma_{\mathrm{Trai}t_{1},\mathrm{Trai}t_{T}} & \ldots & \sigma_{\mathrm{Trai}t_{T},\mathrm{Trai}t_{T}}^{2} \end{array}\right]$$

Based on these predictions of family means, variances and trait-covariances, we can compute the mean of selected family members on the index (*i.e.*, the $UC_{SI}$).

$$UC_{SI}=\mu_{SI}+i_{SI}\times \sigma_{SI}$$

### Including directional dominance as a genome-wide inbreeding effect

Many outbred, clonal crops are known to suffer from inbreeding depression. The typical genome-wide regression models the marker effects as drawn from a normal distribution, with mean zero and an estimated variance parameter. To include directional dominance, we model the genome-wide proportion of loci that are homozygous with the 1×p vector, ***f***, as a fixed-covariate, leading to:

$$y=X\beta +fb+\mathrm{Za}+\Gamma d^{*}+\epsilon .$$

The scalar *b* is the estimated linear effect of overall homozygosity, interpreted as inbreeding depression or heterosis depending on its direction relative to each trait (Xiang *et al.* 2016). The effect of over/under-dominance measured by *b* can be incorporated into the predicted means and variances by dividing *b* by the number of effects (*p*) and subtracting that value from the vector of dominance effects, to get $d=d^{*}-\frac{b}{p}$ (Xiang *et al.* 2016; Varona *et al.* 2018; Werner *et al.* 2020). It is important to note that the partition of genetic effects in this model corresponds to the “biological” (or genotypic) parameterization (Vitezica *et al.* 2013). The dominance coding in the matrix Γ is

$$\begin{array}{c} \gamma_{ij}=\{\begin{array}{cc} \left(0-2p_{j}q_{j}\right) & A_{1}A_{1}\\ \left(1-2p_{j}q_{j}\right) & A_{1}A_{2}\\ \left(0-2p_{j}q_{j}\right) & A_{2}A_{2} \end{array} \end{array}.$$

As a result the effects ***a*** and ***d*** do not correspond to allele substitution and dominance deviation effects directly, but the sum of variance components still equals the $\sigma_{\mathrm{TGV}}^{2}$ and allele substitution effects can be recovered as α=a+d(q−p) in order to predict $g_{BV}$ (Vitezica *et al.* 2013; Varona *et al.* 2018; Werner *et al.* 2020).

### Empirical assessment of the accuracy predicting means, variances, covariances, and usefulnesses in cassava crosses

Since 2012, the Next Generation Cassava Breeding project (http://www.nextgencassava.org) has implemented GS in African and Latin American breeding programs (de Oliveira *et al.* 2012; Ly *et al.* 2013; Wolfe *et al.* 2017). Cassava breeding programs are well-poised to adopt *G_MS_* if, in addition to prediction of means, variances and covariances can be accurately predicted.

### Cassava data: pedigree, genetic map, and phased haplotypes

We chose a publicly available, previously published pedigree, genetic map, and phased marker-dataset as the best starting point for our analysis (Chan *et al.* 2019; https://www.biorxiv.org/content/10.1101/794339v1.full). The pedigree and germplasm chosen represent parents and offspring from the first three cycles of GS conducted at the International Institute of Tropical Agriculture (IITA). These germplasm and genomic selections have been described in greater detail previously (Rabbi *et al.* 2017, 2020; Wolfe *et al.* 2016a,b, 2017, 2019). We note that each cycle of selection was done by recurrent genomic truncation selection using a SI similar (but not identical) to the one described below. The base generation (C0) was the top-ranked clones among a larger collection of the diverse but interrelated elite as well as landrace germplasm. Chan *et al.* (2019) implemented a number of procedures to ensure the quality of the data. First, technical replications of the original genotyping-by-sequencing (GBS) were validated with BIGRED (Chan *et al.* 2018) and reads were combined to reduce missingness and increase read-depth-per-sample. Next, a multi-pass analysis using the pedigree-validation software, AlphaAssign (Whalen *et al.* 2018) was used to ensure only relationships supported by the data were assumed downstream. Genotypes were called using validated pedigree information, and sites with more than 30% missing data were removed, leaving 206,539 out of 336,692 sites (summed across all 18 chromosomes) for analysis. The filtered dataset was used as input for phasing and imputation. Pedigree-guided imputation and phasing were accomplished by SHAPEIT2/duoHMM (O’Connell *et al.* 2014). Finally, the authors constructed a genetic linkage map based on crossover events observed in the dataset. We restricted our analysis to only the 3199 individuals comprising 462 full-sibling families (and their parents), in which both parents were validated/known.

### Cassava data: traits, trials, and selection indices

We chose four focal cassava traits: dry matter percentage (DM), fresh root yield in natural-log tons-per-hectare (logFYLD), season-wide mean cassava mosaic disease severity (1–5 scale; MCMDS), and total carotenoids by color chart (1*–*8 scale; TCHART). These traits include both polygenic (DM and logFYLD) and mono/oligenic architectures (MCMDS and TCHART). Two of the traits are known to have important dominance variance (logFYLD and MCMDS), while DM has been shown to be largely additive (Wolfe *et al.* 2016a,b). From these traits, we composed two hypothetical SI, which represent two real and disparate breeding goals (Supplementary Table S1). Both indices target increased DM and logFYLD and reduced MCMDS. We refer to the first index as the “Standard SI” (or StdSI) as it emphasizes yield and disease resistance in a white-fleshed background. The second index “Biofortification SI” (or BiofortSI) focuses on breaking a historically negative genetic correlation between DM and carotenoid content by weighting most heavily the combination of yellow-flesh (high TCHART) and high DM. We note that the pedigree and germplasm analyzed here arose from genomic truncation selection for the equivalent of the StdSI. For this reason, our population and analyses should not be considered as representative or definitive regarding biofortification breeding goals. We started with unscaled, noneconomic weights and scaled them by dividing by the standard deviation of phenotypic BLUPs (see below) for each trait (Supplementary Table S1).

We used pre-adjusted phenotypes, namely, de-regressed BLUPs as input for our downstream analyses. The field trial data used span from 2013 to 2019 and are available directly from http://www.cassavabase.org. The download, quality control, formatting and mixed-model analysis that produced the BLUPs are fully documented and reproducible here: https://wolfemd.github.io/IITA_2019GS/. The BLUPs produced and used in this study of cross variance prediction were originally used for GS conducted during summer 2019. The entire raw IITA trial download was too large for GitHub and is therefore stored here: https://cassavabase.org/ftp/marnin_datasets/NGC_BigData/.

### Parent-wise cross-validation scheme

We devised a cross-validation scheme that: (1) allowed measurement of the accuracy of predicting means, variances, and covariances in previously unobserved crosses, and (2) enabled us to distinguish accuracy predicting BV from TGV. First, define a vector, ***P*** of the parents listed in the pedigree. Define also a second vector ***C*** listing the genotypes (clones) in the pedigree, including the parents (P⊂C).

We conducted five replications of the following procedure:

1. Define parent-wise cross-validation folds: randomly assign the parents in ***P*** into *k*-folds. We chose *k* = 5 folds or about 42 of 209 parents in ***P*** per fold (defined as $P_{\mathrm{TST}}^{k}$, the list of “test” parents in the *k*th-fold.
2. For each of the *k*-folds (set of 42 “test” parents), divide the clones vector ***C*** into two mutually exclusive sets: “training” ($C_{\mathrm{TRN}}$) and “validation” ($C_{\mathrm{VLD}}$). From the set $C_{\mathrm{TRN}}$, we exclude all descendants (offspring, grandchildren, great grandchildren, etc.) of $P_{\mathrm{TST}}^{k}$. We include the $P_{\mathrm{TST}}^{k}$ themselves (phenotyping the parents before predicting their offspring) and any nondescendents. Define $C_{\mathrm{VLD}}$ simply as the set difference between ***C*** and $C_{\mathrm{TRN}}$.
3. Estimate marker effects independently by fitting mixed-models (see section below for further details) to $C_{\mathrm{VLD}}$ and $C_{\mathrm{TRN}}$ corresponding to each $P_{\mathrm{TST}}^{k}$.
4. For each $P_{\mathrm{TST}}^{k}$, define the set of crosses to predict, $X_{\mathrm{toPred}}^{k}$ to include any of the 462 actual families (sire-dam pairs) in the pedigree, in which the $P_{\mathrm{TST}}^{k}$ were involved. By construction, the real family members that have been observed for each of the $X_{\mathrm{toPred}}^{k}$ were excluded from the model used to get marker effects for $C_{\mathrm{TRN}}$, and included in the model for $C_{\mathrm{VLD}}$. Predict the means, variances and covariances for each focal trait in each cross, $X_{\mathrm{toPred}}^{k}$ using the $C_{\mathrm{TRN}}$ marker effects only.
5. For each family in $X_{\mathrm{toPred}}^{k}$, using all existing family members, compute the sample means, variances, and covariances for **GEBV and GETGV** as predicted by the $C_{\mathrm{VLD}}$ marker effects.
6. Calculate the accuracy of prediction for each mean ($\overset{\mu_{T}}{cor_{BV}},\;\overset{\mu_{T}}{cor_{\mathrm{TGV}}}$), variance ($\overset{\sigma_{t=t}^{2}}{cor_{BV}},\;\overset{\sigma_{t=t}^{2}}{cor_{\mathrm{TGV}}}$) and covariance ($\overset{\sigma_{t\neq t}}{cor_{BV}},\;\overset{\sigma_{t\neq t}}{cor_{\mathrm{TGV}}}$) in terms of both **BV** and ***TGV***. For $\overset{\mu_{T}}{cor}$ we used the Pearson correlation between predicted and sample mean **GEBV/GETGV**. For $\overset{\sigma_{t=t}^{2}}{cor}$ and $\overset{\sigma_{t\neq t}^{2}}{cor}$, only families with greater than two members were able to be included, and we weighted the correlation between the predicted and sample (co)variance of **GEBV/GETGV** according to the family size (R **package**::*function* **psych**::*cor.wt*). For sake of comparison, we also include accuracies in the supplement where predicted values are correlated to phenotypic (rather than genomic-predicted) BLUPs, *e.g.*, ${\overset{\mu_{T}}{cor}}_{BV,\mathrm{BLUP}},\;{\overset{\mu_{T}}{cor}}_{\mathrm{TGV},\mathrm{BLUP}}$, and so on.

The cross-validation scheme is numerically summarized in Supplementary Table S2 (see also Supplementary Tables S3–S5).

### Multi-trait Bayesian ridge regressions

We used the multi-trait Bayesian ridge regression (MtBRR) implemented in the development version of the BGLR R package (https://github.com/gdlc/BGLR-R), which is itself a direct port of the model implemented in the package MTM (de los Campos and Grüneberg 2016). The MtBRR models marker effects as being drawn from a multivariate-normal distribution with mean effects of 0 for each trait and variance-covariance parameters jointly estimated from the posterior distribution of the Gibbs chain. We ran each chain for 30,000 iterations, discarded the first 5000 as burn-in and thinned to every 5th sample. The number of iterations was chosen based on prior univariate analyses using 10,000 iterations (Wolfe *et al.* 2017). Convergence was confirmed visually during initial test runs. We used de-regressed BLUPs as responses in each model to match the approach used for GS (Wolfe *et al.* 2017), but BGLR does not currently support weighted observations in the multi-trait model. Our main focus was on the directional dominance model described above. However, we also fit a nondirectional additive plus dominance model to which we make some comparisons in the Supplementary material.

We fit an MtBRR to each $C_{\mathrm{TRN}}$ and $C_{\mathrm{VLD}}$ as described above. In addition, we analyzed the entire population (“All” samples) and the component genetic groups, which are: **GG** (or **C0**; the original progenitors chosen from a population known as the “Genetic Gain”), **TMS13**, **TMS14,** and **TMS15**, which represent the offspring from 2013 (**C1**), 2014 (**C2**), and 2015 (**C3**), respectively.

### Predicting cross means, variances, and usefulnesses

We predicted cross means using the posterior mean marker effects. For variance predictions, Lehermeier *et al.* (2017a,b) used the posterior mean variance (PMV), which is effectively the mean of the variances predicted by each MCMC-sample of marker effects (see Equations 7–10 in that study). The alternative approach, referred to as the variance of posterior means (VPM), is to make variance predictions simply with the posterior mean marker effects. The PMV is expected to be less biased compared to the VPM but is considerably more computationally intensive. Moreover, PMV requires the on-disk storage of massive posterior marker-effects arrays. We computed the **PMV** for each prediction in the cross-validation study and in estimating population genetic variances. In the Supplementary Appendix, we made a brief comparison of **PMV and VPM** and based on these results, used **VPM** in the exploratory predictions, which are described below. We computed SI means and variances using the predicted (and sample) means, variances and covariances of the component traits, and the index weights, given in Supplementary Table S1.

### Realized selection intensities (measuring post-cross selection)

We used **GEBV and GETGV** based on test-set marker-effects to compute observed (or realized) usefulness criteria *i.e.*, $UC_{\mathrm{parent}}$ and $UC_{\mathrm{variety}}$ and measure prediction accuracy as follows. For $UC_{\mathrm{parent}}$, we computed the mean **GEBV** of family members who were themselves later used as parents. We computed $UC_{\mathrm{variety}}$ using the mean **GETGV** of family members advanced to the penultimate stage of the VDP, the advanced yield trial (AYT). In order to combine predicted means and variances into usefulness criteria, *i.e.*, ${\hat{\mathrm{UC}}}_{\mathrm{parent}}$ and ${\hat{\mathrm{UC}}}_{\mathrm{variety}}$, we first calculated the realized intensity of within-family selection ($i_{RS}$ and $i_{\mathrm{VDP}}$). For $i_{RS}$, we used the proportion of family members who themselves appear in the pedigree as parents. For the $i_{\mathrm{VDP}}$, we used the raw plot-basis data to compute the proportion of clones from each family with at least one plot in the aforementioned AYT stage of the VDP, as of July 2019. We computed standardized selection intensity in R using i=dnorm(qnorm(1−propSel))/propSel, where *propSel* is the proportion selected.

### Exploratory analysis: predictions of previously untested crosses

We conducted a prediction exercise evaluating the interest of possible future crosses compared to those previously made in terms of additive and total merit, *i.e.*, $UC_{\mathrm{parent}}$ and $UC_{\mathrm{variety}}$. We predicted the means and variances of all possible pairwise matings between the union of 209 parents already used and the 100 clones with top rank on the StdSI, of which only 3 overlapped (*N *=* *306 parents). This resulted in 47,083 crosses to predict. We used marker-effects from the full-model (all clones included). We predicted means, variances, and covariances for all four traits and subsequently used these to compute StdSI and BiofortSI means and variances.

The dataset we analyzed does not include all traits or germplasm relevant to the IITA breeding program. For that reason, our results especially regarding the potential benefits of new matings are meant as an example. Assessment of the actual best new matings to make in the ongoing breeding program will rely on a broader analysis.

## Results

Results along with code generating summaries, figures, and related tables are also available as part of the **workflowr** R markdown website (Results, Figures, Supplementary Figures, and Supplementary Tables).

**Pedigree and Germplasm**: There were 3199 individuals in 462 families, derived from 209 parents in our pedigree. Parents were used an average of 31 (median 16, range 1–256) times as male and/or female parents in the pedigree. The mean family size was 7 (median 4, range 1–72). The average proportion of homozygosity was 0.84 (range 0.76–0.93) across the 3199 pedigree members (computed over 33,370 variable SNP; Supplementary Table S14). As expected for a population under RS, the homozygosity proportion increased with each generation with C0, C1, C2, and C3 having homozygosity proportion of 0.826, 0.835, 0.838, and 0.839, respectively (Supplementary Figure S1).

**Cross-validation Scheme:** Across the 5 replications of fivefold cross-validation, the average number of clones was 1833 (range 1245–2323) for training sets and 1494 (range 1003–2081) for testing sets. The 25 training-testing pairs set up an average of 167 (range 143–204) crosses to predict (Supplementary Table S2).

**BLUPs and**  **SI:** The correlation between the two SI (StdSI and BiofortSI; Supplementary Table S1) based on *i.i.d.* (nongenomic) BLUPs of component traits was 0.43 (Supplementary Figure S2). The correlation between DM and TCHART BLUPs was −0.29.

### Accuracy of family mean prediction

**Figure 1:**
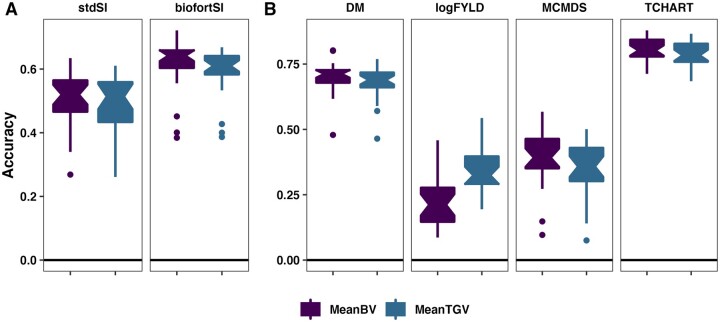
Accuracy predicting family means. Fivefold parent-wise cross-validation estimates of the accuracy predicting the cross means on SI (A) and for component traits (B), are summarized in boxplots. Accuracy (*y*-axis) was measured as the correlation between the predicted and the sample mean GEBV or GETGV. For each trait, accuracies are given for two prediction types: family mean BV *vs* TGV.

Across traits, most accuracy estimates (more than 75%) were lower for prediction of family-mean TGV than for mean BV (median difference TGV-BV = −0.017). The only exception was for yield (logFYLD), where TGV>BV, median increase of 0.13 (Figure 1, Supplementary Table S10). We note that accuracy is higher for BiofortSI compared to StdSI, which makes sense given that BiofortSI emphasizes DM and TCHART, which have higher accuracy than logFYLD and MCMDS.

### Accuracy of within-family variance and covariance prediction

**Figure 2:**
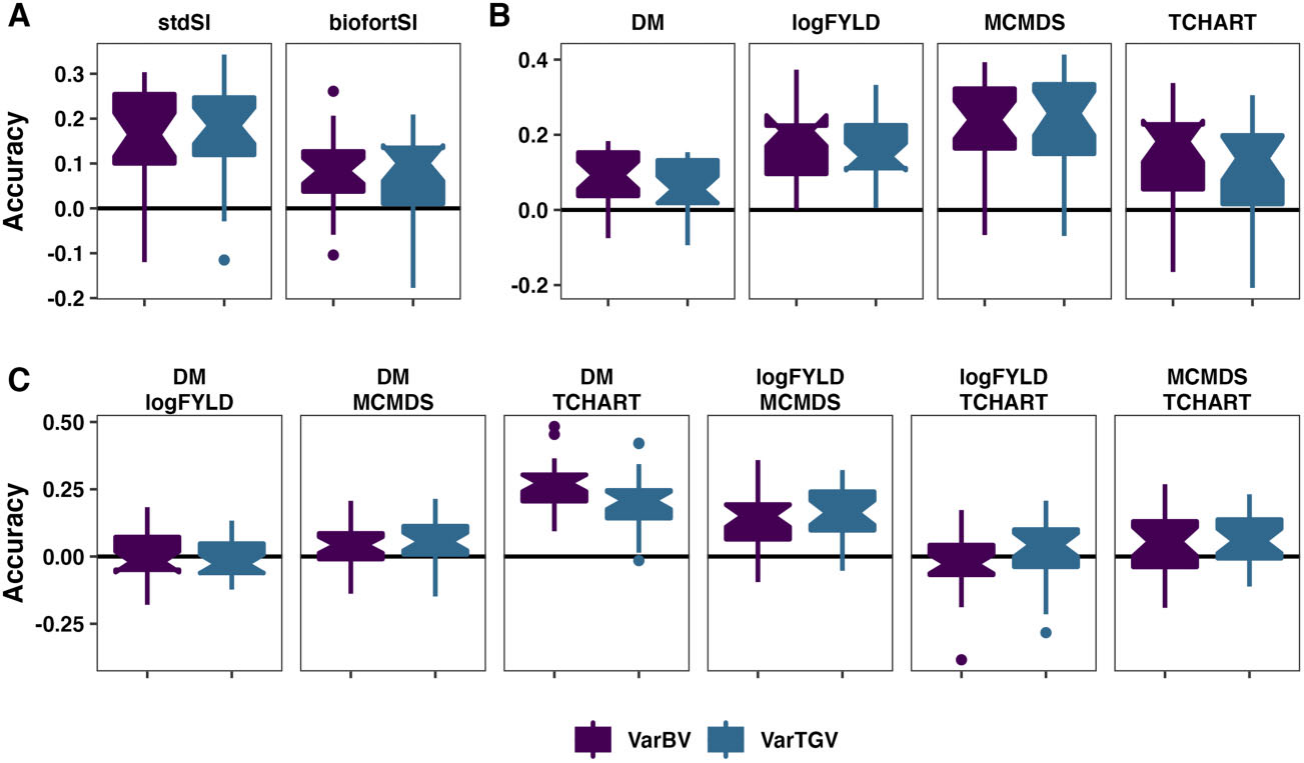
Accuracy predicting genetic (co)variances. Fivefold parent-wise cross-validation estimates of the accuracy predicting the genetic variance of crosses on SI (A) and for component trait variances (B) and covariances (C). Accuracy (*y*-axis) was measured as the correlation between the predicted and the sample (co)variance of GEBV or GETGV. For each trait (panel), accuracies for two prediction types are given: VarBV and VarTGV.

Most (89%) of variance prediction accuracies were greater than zero, with median accuracy 0.14 across traits (Figure 2A, Supplementary Table S11). For covariances, prediction accuracy was lower (median 0.07) and 70% of accuracy estimates were greater than zero (Figure 2B, Supplementary Table S11). In contrast to results for predicting family-means, the most accurately predicted trait-variances were MCMDS, TCHART, and logFYLD with median accuracies (proportion accuracies ≥ 0) of 0.25 (0.92), 0.17 (0.84), and 0.15 (1.0), respectively. Var(DM), for example, had among the lowest median accuracies at 0.07. Interestingly, the DM-TCHART covariance was also very well predicted with median accuracy 0.23 (97% of accuracies ≥ 0). Accuracy for the SI variances were intermediate with median StdSI accuracy = 0.17 (0.92) and BiofortSI = 0.09 (0.78) compared to the component traits. Like the SI accuracy for family-means, accuracy for variances was related to the accuracy of the component traits. In contrast to predicting SI cross-means, for variances, the StdSI ¿ BiofortSI. This makes sense as the StdSI emphasized logFYLD and MCMDS, whose variance were better predicted than those of DM, TCHART, and related covariances. There were, overall, only small differences in accuracy between $\sigma_{BV}^{2}$ and $\sigma_{\mathrm{TGV}}^{2}$ with the median difference being −0.003.

### Accuracy predicting the usefulness of crosses

The observed **UC** are the mean **GEBV** or **GETGV** of family members who were themselves later used as parents or advanced on the VDP. In order to compute the **UC**, we first calculated the realized intensity of within-family selection ($i_{RS}$ and $i_{\mathrm{VDP}}$) (Supplementary Figure S3; Supplementary Table S13). There were 48 families with a mean intensity of 1.59 (mean 2% selected) that themselves had members who were parents in the pedigree and could be used to validate $UC_{\mathrm{parent}}$ predictions. There were 104 families for validation of $UC_{\mathrm{variety}}$ predictions, with mean intensity 1.46; mean 5% members selected and advanced to the AYT stage of the VDP. On a per-repeat-fold basis, the number of families with observed usefulness for measuring prediction accuracy was limited. For $UC_{\mathrm{parent}}$ there were an average of 17 families (min 9, max 24). For $UC_{\mathrm{variety}}$ the sizes for the focal AYT stage of the VDP were an average depended on the VDP stage, for the focal stage $UC_{\mathrm{variety}}^{\left[\mathrm{AYT}\right]}$, mean number of 37 families was 37 (min 25, max 50) per-repeat-fold.

**Figure 3:**
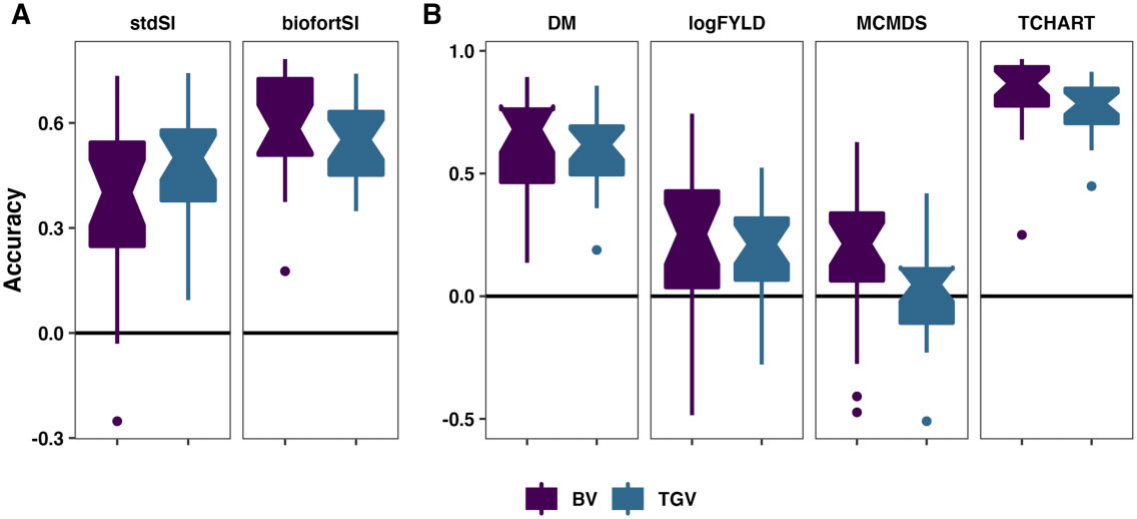
Accuracy predicting cross usefulness (the expected mean of future *selected* offspring). Fivefold parent-wise cross-validation estimates of the accuracy predicting the usefulness of crosses on the SI (A) and for component traits (B), are summarized in boxplots. Accuracy (*y*-axis) was measured as the family-size weighted correlation between the predicted and observed usefulness of crosses for breeding parents ($UC_{\mathrm{parent}}$) or varieties ($UC_{\mathrm{variety}}$).

Most estimates (95%) of UC accuracy were greater than zero, with per-trait accuracies largely similar to the family-mean predictions. Indeed, the overall correlation between mean and UC accuracies was 0.75. As might be expected, given the incorporation of variance-predictions and the more limited validation sample size, the UC accuracy was on average lower by −0.09 compared to the family-mean accuracy (Figure 3, Supplementary Table S12). In contrast to predictions of cross variances, the median UC for the BiofortSI was higher (0.58) compared to the StdSI (0.49). Among component traits, median accuracy ranks TCHART (0.83) > DM (0.65) > logFYLD (0.24) > MCMDS (0.10). As with the mean, there was a tendency (62% of estimates) for $UC_{\mathrm{parent}}$ to be slightly better predicted than $UC_{\mathrm{variety}}$ (median magnitude of difference = −0.06). Prediction accuracy for ***UC*** was similar when setting a constant intensity of 2.67 instead of using family-specific realized intensity (Supplementary Table S12).

### Population estimates of the importance of dominance variance

**Figure 4:**
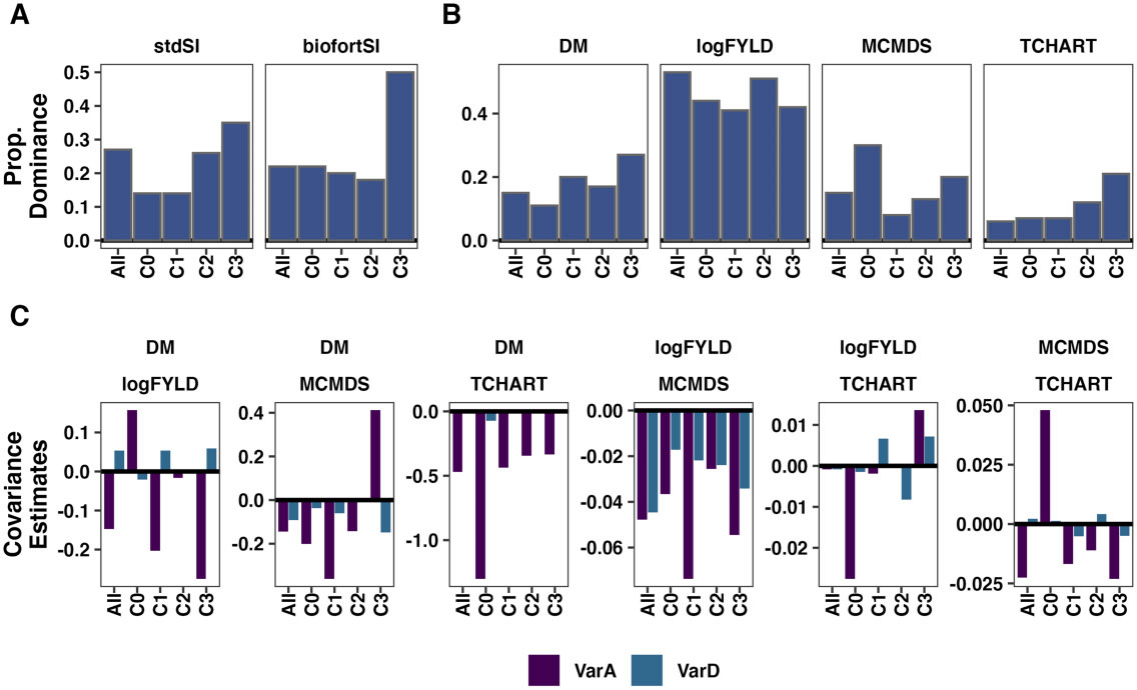
Population-level measures of the importance of dominance genetic effects. The genetic variance estimates from the models fitted to the overall population (“All”) and also to its four genetic groups (*x*-axis) are presented in these barplots. Each panel contains results for a trait variance or covariance. For SI (A) and component traits (B) the proportion of genetic variance accounted for by dominance is shown on the *y*-axis. For covariances between component traits (C) the estimates themselves are plotted. In C, fill color indicates variance component (additive *vs* dominance).

Our focus is mainly on distinguishing among crosses, and the accuracy of cross-based predictions. Detailed analysis of the additive-dominance genetic variance-covariance structure in cassava (sub)-populations is an important topic, which we mostly leave for future study. We make a brief examination of the genetic variance-covariance estimates associated with the overall population and component genetic groups. We report all PMV-covariance estimates in Supplementary Table S15 and complete BGLR output in the repository associated with this study. We focus here on genetic variance-covariance accounting for LD, as in Lehermeier *et al.* (2017a), “Method 2.” Over all genetic groups analyzed, across trait and SI variances, dominance accounted for an average of 24% (range 6–53%). Dominance was most important (mean 46% of genetic variance) for yield (logFYLD) and least important for TCHART (mean 11%) (Figure 4). For several estimates, there was an opposing sign between additive and dominance components, *e.g.*, positive dominance but negative additive genetic covariance for DM-logFYLD.

### Population estimates of inbreeding effects

**Figure 5:**
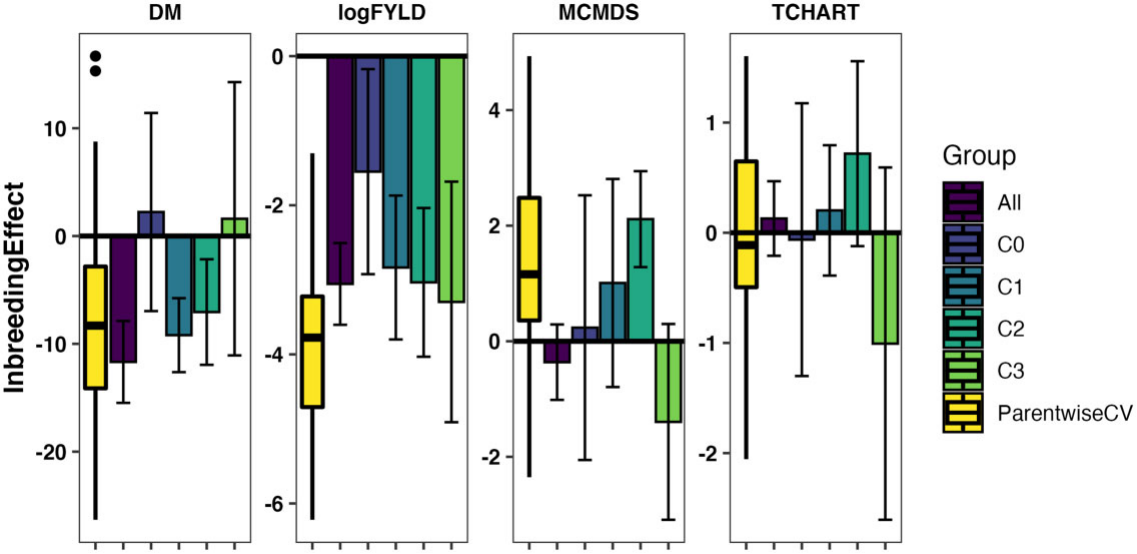
Estimates of the genome-wide effect of inbreeding. For each trait (panels), the fixed-effect for genome-wide proportion of homozygous sites is shown on the *y*-axis, as estimated by a directional dominance model. For the overall population (“All”) and four genetic groups (“C0” C1”C2”C3”), the posterior mean estimate and its standard deviation (bars) are shown on the *x*-axis. For comparison a boxplot showing the distribution of estimates from models fit to parent-wise cross-validation training and validation sets (“ParentwiseCV”) is also shown.

We found that genome-wide estimates of the effect of homozygosity were consistently negative for logFYLD with a mean directional dominance regression coefficient of −2.75 log(tons/ha) across genetic groups (mean effect −3.88 across cross-validation folds). In addition, DM estimates indicated inbreeding depression effects in several genetic groups and the majority of cross-validation folds with mean directional dominance regression coefficient of −4.82 percent dry matter across genetic groups (mean effect −7.85 across cross-validation folds) and similarly for MCMDS, mean inbreeding effect of 0.32 worse disease severity across genetic groups (mean effect 1.27 across cross-validation folds). This corresponds to higher homozygosity being associated with lower DM, lower yield, and greater disease severity (Figure 5, Supplementary Table S16).

### Exploring predictions about untested crosses

**Figure 6:**
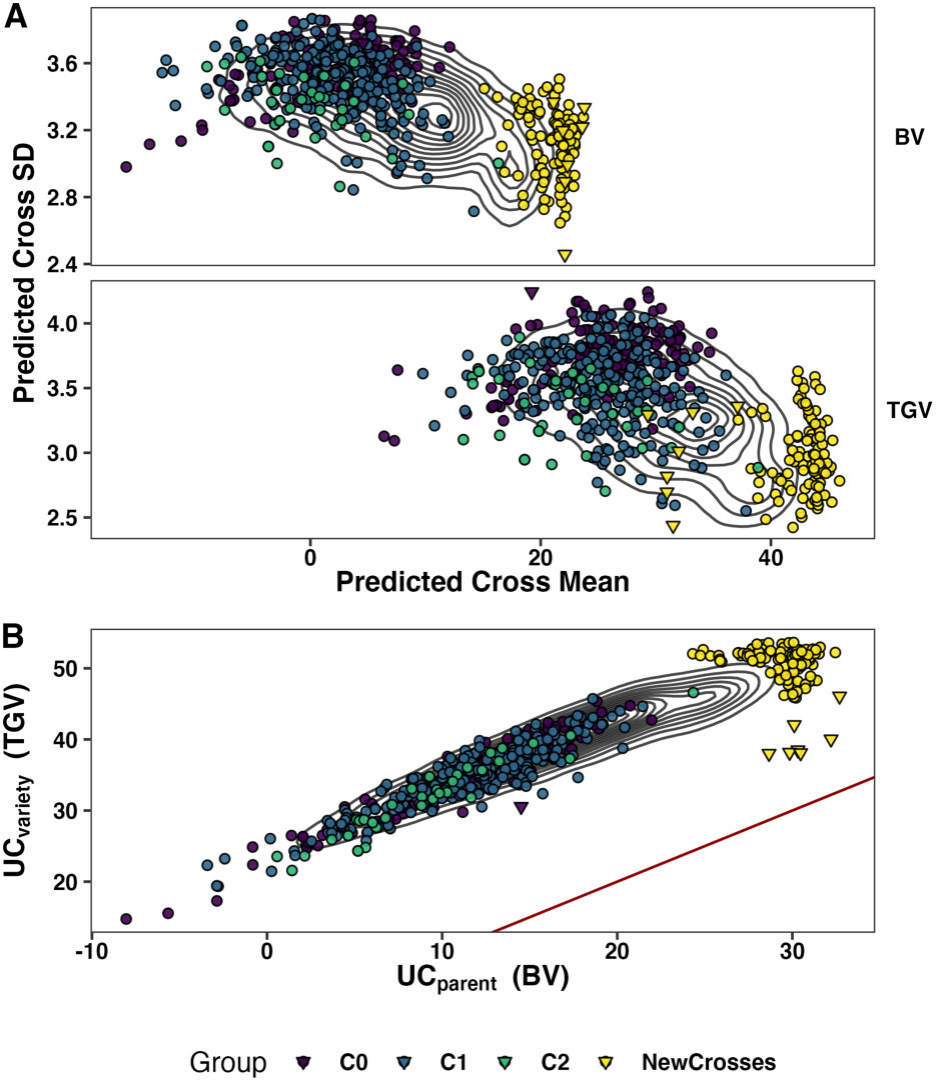
Genomic mate selection criteria for the StdSI predicted for previously untested crosses. We predicted 47,083 crosses among 306 parents. We made four predictions: 2 variance components [BV, TGV] × 2 criteria [Mean, UC = Mean + 2*SD]. Two-dimensional contour lines show the distribution of all predicted crosses. For each of the predictions, we took the top 50 ranked crosses and then selected the union of crosses selected by at least one metric. The 462 crosses previously made are also shown and genetic groups (C0, C1, and C2) are distinguished by color from the 112 new crosses to highlight the opportunity for improvement. Selfs are shown as triangles, outcrosses as circles. The predicted cross genetic mean is plotted against the predicted family genetic standard deviation (Sd, *σ*) for BV and TGV (panel rows) (A). The *UC_parent_* is plotted against the *UC_variety_* with a red one-to-one line in B.

We made 8 predictions (2 SIs × 2 selection targets [BV, TGV] × 2 criteria [Mean, UC = Mean + i*SD]) for each of 47,083 possible crosses of 306 parents. We examined the correlation structure among these predictions in order to understand the multivariate decision space they describe (Figure 6, Supplementary Figures S4 and S5).

The two SI are (by design) disparate breeding goals. The mean correlation (across var. components) between SIs was low for predictions of the family mean (0.20) and lower for the UC (0.14), but high for the SD (0.91). The predictions of BV and TGV were strongly correlated with 0.95 (0.96) for predicted cross means on the StdSI (BiofortSI), 0.88 (0.91) for predicted genetic standard deviation, and 0.93 (0.95) for UC.

The predicted cross means and variances had a low, but negative correlation (Figure 6A). Across traits and variance components, the average correlation between predicted mean and standard deviation ($\overset{\mu ,\sigma }{cor}$) was −0.37. At the standardized intensity of 2.67 (1% selected) the predicted UC was dominated by the mean (average $\overset{\mu ,UC}{cor}$ = 0.995) and there was a small negative correlation between variance and UC (average $\overset{\sigma ,UC}{cor}$ = −0.26).

We wanted to know how selections of crosses-to-make would be affected by our choice of criteria. Separately, for each of the 8 predictions of 47,083 crosses, we selected the top 50 ranked crosses (Supplementary Table S19). In total, only 202 unique crosses were selected based on their rank on at least one of the 8 predictions. Of those, 112 were selected for the StdSI (90 Biofort) and included only 7 (6) self-crosses. No crosses were selected for both SI. None of the selected crosses have previously been tested in the IITA breeding program. We plotted the predicted *μ vs* the *σ* **(**Figure 6A**)** and the *UC_parent_ vs* the *UC_variety_* (Figure 6B). We highlight the unique new crosses proposed and contrast them to the 462 previously made, distinguishing genetic groups (selection-cycle-of-origin, C0, C1, and C2) by colors, in order to illustrate the extent to which our genomic mate selection criteria propose novel and putatively better crosses. For simplicity, we plotted predictions for StdSI only.

**Figure 7:**
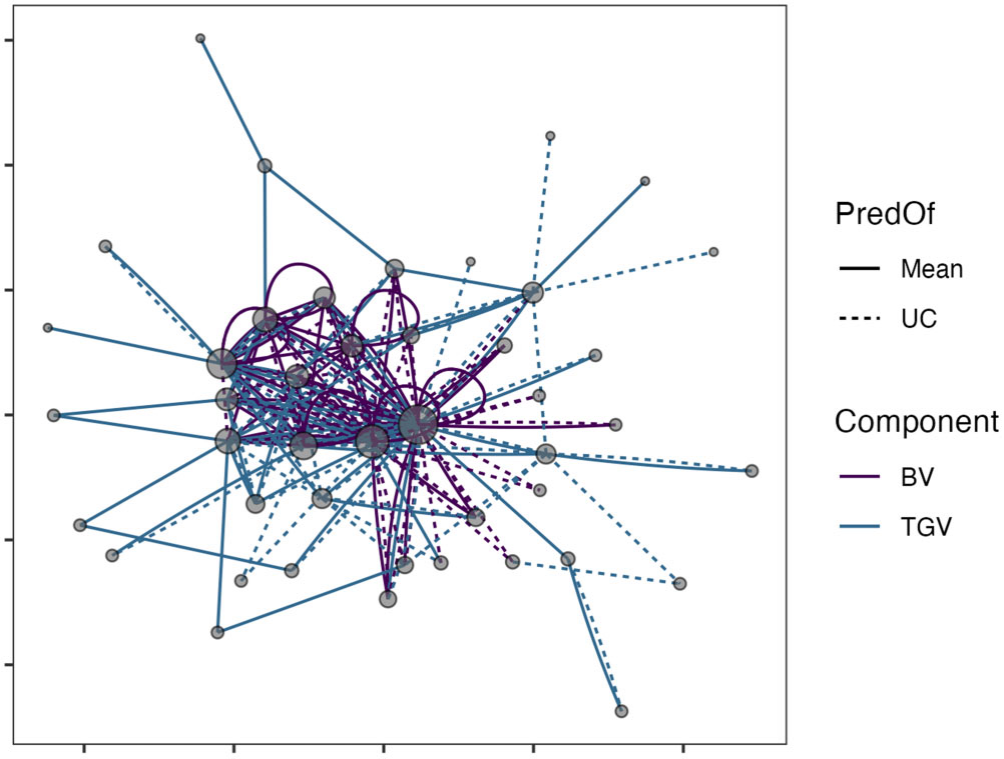
Network plot of selected parents and matings for the StdSI. There were 44 parents and 112 crosses chosen because they were in the top 50 for at least one of four predictions: 2 variance components [BV, TGV] × 2 criteria [Mean, UC = Mean + 2*SD]). Parents are shown as nodes, with size proportional to their usage (number of connections). Matings are shown as edges, with linetype distinguishing selection based on Mean (solid) and UC (dashed) and color depicts selection for BV *vs* TGV.

There were 44 parents represented among the 112 “best” crosses for StdSI with a median usage in 3 families each (range 1–70, most popular parent = **TMS13F1095P0013**). Only 33 parents among 90 “best” crosses were indicated for the BiofortSI with a median contribution to 4 (range 1–81, most popular parent = **IITA-TMS-IBA011371**) crosses. Figure 7 breaks down the selections on the StdSI according to prediction and variance components as a network where selected parents are nodes and matings are edges. For the StdSI, only 17 of 112 crosses (30 of 90 for BiofortSI) were selected jointly for *both* BV and TGV. Self-crosses were only selected based on BV. In fact, 22 of 44 parents selected on the StdSI were chosen only for the TGV of their crosses and 4 only for their BVs (Figure 7). For the BiofortSI, one parent was chosen only for BV, but 14 of 33 were only interesting for their TGV. Only 27 crosses for the StdSI (14 for BiofortSI) were selected *only* based on the UC (*i.e.*, selected for their variance *but not* their mean). As judged by the number of times a cross was chosen given the four selection criteria, there are 58 unique crosses in the top 50 for the StdSI and 66 for the BiofortSI. This demonstrates a relatively simple approach for selecting the overall best crosses based on the four predicted mate selection criteria.

## Discussion

We developed and tested genomic mate selection criteria suitable for multi-trait index selection in organisms of arbitrary homozygosity level where the *F*_1_ (full-sibling progeny) are of direct interest as future parents and/or cultivars (varieties). We focused on the prediction of the SI-associated genetic variance of crosses based on the haplotypes of proposed parents, estimates of marker effects, and estimates of recombination frequencies between marker loci. We combined the predicted mean and variance of a cross into usefulness criteria for parent ($UC_{\mathrm{parent}}$) and variety ($UC_{\mathrm{variety}}$) development, by predicting the genetic variance of both breeding ($\sigma_{BV}^{2}$) and TGVs ($\sigma_{\mathrm{TGV}}^{2}=\sigma_{BV}^{2}+\sigma_{DD}^{2}$).

### Sufficiency and implications of prediction accuracy estimates

We worked with 462 real cassava families of heterogeneous size. We made practical use of the available data in implementing the parent-wise cross-validation scheme. We found that prediction accuracy for family means were largely similar to our previously published estimates (Wolfe *et al.* 2016a, 2017). Variance and UC prediction accuracies were lower than mean prediction accuracies in general. Given that variances are the second-moment of the distribution, it makes sense that accuracies for variances are lower than for means (Zhong and Jannink 2007; Osthushenrich *et al.* 2018; Neyhart and Smith 2019). The accuracy predicting the family mean for a given trait was not well correlated with the accuracy estimate for predicting family variances (*r* = −0.22). This suggests that, for a given phenotype, breeding programs cannot simply rely on available estimates of family-mean prediction accuracy to determine the adequacy of family-variance predictions. The UC and family-mean accuracy estimates were reasonably correlated (*r* = 0.75).

Many factors contribute to achieving optimal accuracy and those factors are well understood in the literature. We focused here on getting an assessment of the overall ability to distinguish crosses with high *vs* low genetic variances. Previous studies of variance-prediction accuracy evaluated relatively few families, but with larger size (Osthushenrich *et al.* 2018; Yao *et al.* 2018; Neyhart and Smith 2019). Interestingly, we found that traits with the most accurately predicted variances had less accurately predicted means, including the SIs (mean: StdSI < BiofortSI; variance: StdSI > BiofortSI; Figures 1 and 2). This does not seem initially explainable by our priors regarding trait genetic architectures; DM and FYLD are both generally considered as polygenic/infinitesimal traits, while MCMDS and TCHART are expected to be closer to mono- or oligogenic (Wolfe *et al.* 2016b;Rabbi *et al.* 2020). Differences in accuracy between mean and variance predictions should instead have to do with the nature of linkage disequilibrium, especially as it affects marker-causal relationships (de Los Campos *et al.* 2015; Lehermeier *et al.* 2017a). Similar to the simulations and empirical results of Neyhart *et al.* (2019) we found that the DM-TCHART covariance was particularly well predicted, corresponding to hypothesized tight linkage between QTL on chromosome 1 (Rabbi *et al.* 2017); a region known to contain large low-recombination regions of historical introgression from the wild relative *M. glaziovii* (Wolfe *et al.* 2019).

The actual accuracy achieved should be higher than our estimates. Our empirical estimates of progeny variance, against which we validate our predictions, are subject to both Mendelian sampling and effect estimation error. That error decreases the correlation, biasing all our estimates downward. Put another way, we make predictions of the variance of an infinite number of progeny, but are only able to correlate those predictions to a real sample of families with finite and heterogeneous numbers of offspring.

Several conditions for the implementation of cross-variance predictions and mate selection need to be met. First, predictions of GEBV or GETGV are considered suitable for genomic truncation selection; for example, based on cross-validation and/or cross-generation prediction accuracy estimates. Second, genetic maps are established and trusted. Finally, accurate marker data phasing for candidate parents must be available. If these criteria are met the logistics of mate selection are feasible.

The possibility remains that estimates of variance might be poor enough to contribute more noise than signal to the selection decisions. The answer is hard to intuit and decisions must be made on a program-specific basis. By obtaining a prediction of cross-variance we add a component of information to the cross-mean predictions we had before. We also add a potential source of error. One suggestion might simply be to incorporate cross-variance predictions into selections via the UC cautiously by choosing a relatively low-standardized selection intensity value when incorporating the mean and variance predictions. Field validating variance predictions across multiple large families and simulating long-term outcomes may offer the best viable additional sources of decision support regarding the use of usefulness predictions.

### The importance of nonadditive effects and the effect on inbreeding

Nonadditive effects are important in cassava, accounting for an average of 24% of genetic variance in this study. Our results are consistent with previous studies that highlight the importance of nonadditive effects for fresh root yields but not for dry matter or total carotenoid content (Esuma *et al.* 2016; Wolfe *et al.* 2016a, 2017; Nduwumuremyi *et al.* 2018; Andrade *et al.* 2019). To our knowledge, we are the first to report partitions of trait-trait genetic *co*variance into additive and dominance components, though we do not comment on it in detail in this study. We also report the first estimates of genome-wide marker-based directional dominance in cassava. Using the model of Xiang *et al.* (2016), we found notable evidence of inbreeding *depression*, for every trait except TCHART, but especially yield. Our results match several previous estimates of inbreeding depression based on field observation of selfed (S_1_) progeny (Pujol and McKey 2006; Rojas *et al.* 2009; Kawuki *et al.* 2011; de Freitas *et al.* 2016). Theory and data (reviewed in Kristensen and Sørensen 2005) indicate that traits more closely associated with fitness (in cassava, this would be traits related to root and stem production, for example) should be more impacted by directional dominance (inbreeding depression). These results also make sense in light of the evidence of deleterious genetic load in cassava (Ramu *et al.* 2017) and balancing selection for heterozygosity in introgression regions (Wolfe *et al.* 2019). It was also interesting to observe that there are unique matings with elite rank only for TGV (nonadditive effects) and still others uniquely interesting for BV. The network plot in Figure 7 shows a pattern of crosses within *vs* among particular parents selected for exploiting either BV or TGV that warrants future investigation. Our cross-validation scheme allows us to distinguish TGV and BV accuracy by using genomic estimates of BV and TGV as validation data. Nevertheless, we found only small differences between TGV and BV accuracy for both mean and variance-related predictions. The composition of a mating plan based on *UC_parent_ and UC_variety_* is still an important decision point for breeders. To a certain extent, choices depend on a breeder’s emphasis on matings to produce varieties *vs* to improve the population overall. In the future, numerically optimized mating plans that balance investment in crosses to maximize the value of parents *and* varieties produced by each crossing block can be developed.

### Caveats, limitations, and future directions for GMS in outbred, clonal crops

In some circumstances, for computational efficiency, it may be desirable to use the VPM rather than the PMV approach to predict cross variances. Our results show that the correlation between VPM and PMV predictions is very high *but* their magnitude is different, as is the accuracy estimate (see Supplementary Appendix). If any bias is consistent, then ranking differences between PMV and VPM (or REML) predictions of cross variance will not occur. Ultimately, if implementing mate selections via the usefulness criteria, choosing the VPM method would mostly have the consequence of shrinking the predicted values toward the mean.

Other critical considerations for practical implementation include the necessary phasing quality and method. We leveraged a dataset imputed and phased using a validated pedigree (Chan *et al.* 2019); many plant breeding programs may not have suitable pedigree or depth of relationships to enable this. We do not rule out using “standard” population-based imputation and phasing (*e.g.*, Browning and Browning 2016). Promising also will be the development of a practical haplotype graph suitable for outbred diploids like cassava (Jensen *et al.* 2020; Zou *et al.* 2020). In addition, the necessary marker density for accurate prediction should be considered as it has a very significant effect on computational speed.

Several extensions and future directions are of interest moving forward from the current study. We have only addressed dominance, but extensions of variance prediction to include epistasis or even nonlinear kernel types should be straightforward (Alves *et al.* 2019). The directional dominance model and its assumption of uncorrelated additive and dominance effects and linear genome-wide effects on phenotype of increasing homozygosity need evaluation (Xiang *et al.* 2018). We note that *many* outbred, clonal crops are actually polyploids. For organisms with such genomes, further developments in recombination mapping, phasing, and prediction models will be required, but are expected to be possible. In our study, we focused on trait-associated variance prediction. Considerable development of mate selection criteria has concerned the avoidance of genetic diversity loss *generally*, these are approaches that constrain inbreeding (Kinghorn 2011; Woolliams *et al.* 2015) and are distinct from trait-associated predictions presented here. We note that Allier *et al.* (2019b) recently described prediction of the variance in parental contribution in a family (*i.e.*, the variance in inbreeding level) as a correlated trait, using an extension of the approach for prediction trait-associated variance.

## Conclusions

By providing predictions of the selection-index-associated means *and* variances in arbitrary crosses for additive *and* dominance variances, we provided a suite of genomic *mate* selection criteria suitable for the complexities of a modern (cassava) breeding program. We presented a simple approach for genomic truncation mate selection that identifies a profile of crosses collectively interesting because of the predicted merit of their progeny in terms of$\mu_{BV},\;\mu_{\mathrm{TGV}},\;\sigma_{BV}$, and $\sigma_{\mathrm{TGV}}$. Ultimately, crossing plans can be numerically optimized (Akdemir and Sánchez 2016; Gorjanc and Hickey 2018; Akdemir *et al.* 2019; Allier *et al.* 2019a) to consider trait-associated means and variances as well as inbreeding levels, to provide a high degree of control for the management of breeding populations.

## Data availability, reproducibility, and predCrossVar R package

We accessed the pedigree, genetic map and haplotypes from the Cassavabase FTP server repository for Chan et al. 2019: ftp://ftp.cassavabase.org//manuscripts/Chan_et_al_2019. The full repository for this study including all data and output can also be accessed through the Cassavabase FTP server by choosing "Guest" credentials, here: ftp://ftp.cassavabase.org//manuscripts/Wolfe_et_al_2021. Alternatively, all files are also available through the Cassavabase FTP server-archive: https://cassavabase.org/ftp/manuscripts/Wolfe_et_al_2021/. The repository, minus large data files, can be found on GitHub, here: https://github.com/wolfemd/PredictOutbredCrossVar/. We used Rmarkdown and the R package workflowr (version 1.6.2, https://github.com/jdblischak/workflowr) to document our empirical analysis as a fully reproducible website: https://wolfemd.github.io/PredictOutbredCrossVar/. Finally, we implemented the core functions for multi-trait prediction of outbred cross variances including additive and dominance effects in an R package **predCrossVar**, repository on GitHub: https://github.com/wolfemd/predCrossVar}, package reference manual: https://wolfemd.github.io/predCrossVar/, which we used in the aforementioned analyses. Supplemental Material available at figshare: https://doi.org/10.25386/genetics.14569044.
