# Corrigendum to: Genomic mating in outbred species: predicting cross usefulness with additive and total genetic covariance matrices

**DOI:** 10.1093/genetics/iyab225

**Published:** 2021-12-30

**Authors:** Marnin D Wolfe, Ariel W Chan, Peter Kulakow, Ismail Rabbi, Jean-Luc Jannink

*Genetics*, 2021, https://doi.org/10.1093/genetics/iyab122

In the originally published version of this manuscript, several equations were improperly formatted, thus not conveying information accurately to readers. Additionally, some hyperlinks were omitted. These errors have been corrected.

